# Intra‐rater reliability of leg blood flow during dynamic exercise using Doppler ultrasound

**DOI:** 10.14814/phy2.15051

**Published:** 2021-10-07

**Authors:** Sachin B. Amin, Hendrik Mugele, Florian E. Dobler, Kyohei Marume, Jonathan P. Moore, Justin S. Lawley

**Affiliations:** ^1^ Department Sport Science Division of Performance Physiology and Prevention University Innsbruck Innsbruck Austria; ^2^ School of Sport, Health & Exercise Science Bangor University Bangor UK

**Keywords:** Doppler ultrasound, muscle blood flow, reliability, whole‐body exercise

## Abstract

Developing an exercise model that resembles a traditional form of aerobic exercise and facilitates a complete simultaneous assessment of multiple parameters within the oxygen cascade is critically for understanding exercise intolerances in diseased populations. Measurement of muscle blood flow is a crucial component of such a model and previous studies have used invasive procedures to determine blood flow kinetics; however, this may not be appropriate in certain populations. Furthermore, current models utilizing Doppler ultrasound use isolated limb exercise and while these studies have provided useful data, the exercise model does not mimic the whole‐body physiological response to continuous dynamic exercise. Therefore, we aimed to measure common femoral artery blood flow using Doppler ultrasound during continuous dynamic stepping exercise performed at three independent workloads to assess the within day and between‐day reliability for such an exercise modality. We report a within‐session coefficient of variation of 5.8% from three combined workloads and a between‐day coefficient of variation of 12.7%. These values demonstrate acceptable measurement accuracy and support our intention of utilizing this noninvasive exercise model for an integrative assessment of the whole‐body physiological response to exercise in a range of populations.

## Graphical abstract

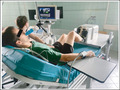

**New Findings**

**What is the central question of this study?**

Determine reliability of measuring skeletal muscle blood flow during dynamic continuous exercise using Doppler ultrasound.

**What is the main finding and its importance?**

We demonstrated that muscle blood flow could be reliably measured within and between days to acceptable standards using Doppler ultrasound at three different workloads during continuous dynamic stepping exercise. Such a model, combined with assessment of multiple parameters within the oxygen cascade facilitates a complete integrative approach for determining exercise intolerance in multiple populations.

## 1 INTRODUCTION

The oxygen cascade refers to the diffusion and transport of oxygen through the pulmonary and systemic circulations culminating in mitochondrial respiration at the level of the skeletal muscle. Delivery and flow of oxygen within skeletal muscle vasculature is an essential component of the oxygen cascade and is reduced in elderly (Proctor et al., 1998) and diseased populations (Houstis et al., 2018), contributing to exercise intolerance. Previous studies examining blood flow kinetics during dynamic exercise have typically used invasive techniques such as thermodilution (González‐Alonso & Calbet, 2003), which provide valuable data in healthy populations, however may be unsuitable for examining exercise intolerances in elderly, diseased, or adolescent populations.

Over the last few decades, ultrasonography has gained popularity as a noninvasive technique to quantify muscle blood flow (Guirro et al., 2017). Indeed, ultrasonography has several other advantages beyond being non‐invasive such as providing high‐resolution real‐time measurements and the capacity to quantify antegrade and retrograde shear rates, which are intimately linked to vascular adaptation and/or dysfunction (Green et al., 2017). However, artifacts due to the form of dynamic movements (i.e., large displacement of arteries caused by muscle contraction and extraneous whole‐body movement causing probe movement artifacts) have restricted the variety of exercise modalities that can be studied. To date, the primary exercise modality has been varying forms of the isolated rhythmic forearm (Amundsen et al., 2002; Shoemaker et al., 1996) or knee extensor contractions (Rådegran, 1997; Shoemaker et al., 1994; Walther et al., 2006). These exercise modalities have several advantages in demonstrating the time course of hyperemia post contraction, identifying blood flow kinetics during periods of muscle contraction and /or relaxation (Tschakovsky et al., 2004) and due to the small, activated muscle mass, vasodilator capacity largely independent of central cardiac limitations (Magnusson et al., 1997).

Nonetheless, while these aforementioned exercise models provide valuable insights into skeletal muscle blood flow kinetics, the overall hemodynamic response substantially differs from more traditional forms of lower limb exercises such as walking, running, and cycling, where improvements in exercise capacity or exercise intolerance are reported. For example, running and cycling are characterized by shorter duration muscle contractions and lower force output per stride or leg rotation compared to knee extensor exercise (Rådegran, 1997), which ultimately leads to differences in beat‐by‐beat variability in blood flow (Erikson et al., 1990). Moreover, unlike forearm contractions or leg kicking, traditional lower limb dynamic exercise leads to (1) an increase in cardiac filling pressures due to activation of the muscle pump and the subsequent increase in stroke volume (Katayama et al., 2014), (2) a large reduction in total peripheral resistance (Laughlin, 1999) and dependent on intensity, (3) an increase in sympathetic activity (Katayama & Saito, 2019) causing an increase in resistance within non‐active vascular beds (Hansen et al., 2020).

Therefore, the aim of the present investigation was to test the applicability of an exercise model that closely resembled traditional forms of lower limb exercise and was amenable to assessing each step of the oxygen cascade over mild (hyperemia independent of cardiac limitations) to moderate intensity workloads. Important considerations for the model were: (1) skeletal muscle blood flow could be measured with a low within and between‐day reliability and validity (i.e., a linear relationship between leg blood flow and oxygen consumption (2) exercise could be performed in the upright or at least semi‐recumbent posture. This latter point is important for future studies aiming to incorporate an integrative model to identify cardiac, cerebral, or skeletal muscle vascular responses to exercise, as gravity has a profound effect on venous return to the heart (Laughlin, 1999), the collapsibility of jugular veins and thus cerebral perfusion pressure (Lawley et al., 2017; Qvarlander et al., 2013) and skeletal muscle vasodilator responses.

## 2 METHODS

### 2.1 Participants

Thirteen male and four female participants (28 ± 3.5 years, height, 178.3 ± 9.3 cm, weight 76.2 ± 11.9 kg) were recruited via personnel communication from the University of Innsbruck. All participants were healthy, nonsmokers, and free from cardiovascular, metabolic, and neuromuscular diseases. Female participants were tested during the early follicular phase, or placebo phase of their menstrual cycle if on contraceptives (pill; two). Written informed consent was provided for all participants following detailed verbal explanations of the experimental protocol. The study conformed to the standards set out by the Declaration of Helsinki, except for registration in a database, and was approved by the ethics committee of the University of Innsbruck (34/2018).

### 2.2 Experimental design

All trials were completed in a quiet physiology laboratory at the University of Innsbruck. To ascertain the validity and reliability of ultrasound to quantify muscle blood flow during dynamic exercise, participants performed semi‐recumbent stepping exercise at three workloads on two occasions separated by 1 week. The workloads were targeted at an absolute oxygen uptake (VO_2_) of 0.8 l·min^−1^ (workload one—WL1), 1.2 l·min^−1^ (workload two—WL2), and 1.6 l·min^−1^ (workload three—WL3). The workloads were chosen to encompass a range of activities of daily living and the interactive effect of central and peripheral limitation to exercise (Jetté et al., 1990). (1) ~2–4 metabolic equivalents (METs): isolated skeletal muscle hyperemia with no central limitations. (2) ~5–8 METs: mild exercise involving local skeletal muscle hyperemia without large changes in systemic hemodynamics or elevated sympathetic activity (Saito et al., 1997) (3) ~8–12 METs: Moderate intensity exercise involving systemic hyperemia with noteworthy increases in sympathetic nerve activity, vasoconstriction within non‐active skeletal muscle, increased stroke volume, cardiac output, and arterial blood pressure (Saito et al., 1993). Each exercise bout lasted between 5 and 8 min. Stepping cadence was fixed to 80 steps per minute for all workloads and guided by a metronome.

### 2.3 Protocol

**FIGURE 1:**
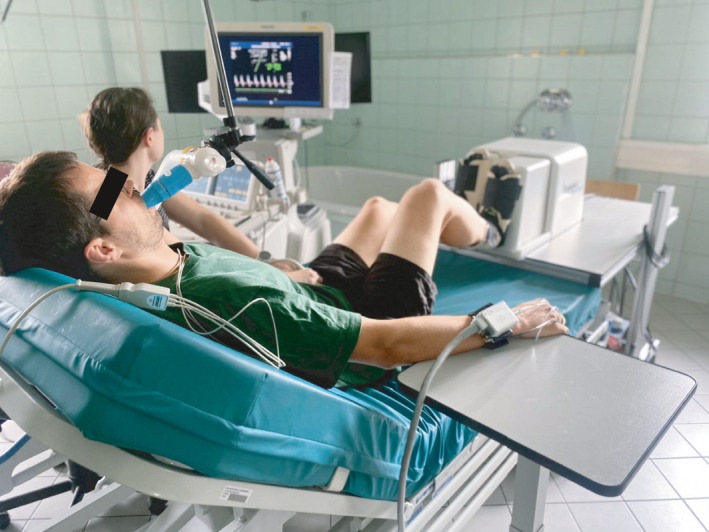
Experimental set up of a participant performing stepping exercise during measurement of femoral artery blood flow with Doppler ultrasound

Participants were instructed to refrain from strenuous exercise (24 h), avoid the consumption of caffeine (12 h) and foods high in nitrates (list provided) (12 h). Additionally, all tests were conducted at the same time of day. Upon arrival, height (Stadiometer, US) and body mass (Kern DS 150k1, Kern & Sohn, Germany) were recorded and participants positioned themselves on a semi‐recumbent bed, which had a cardio stepper (Ergospect medical technology, Innsbruck, Austria) attached. Both arms were abducted to the side and rested at heart level with a blood pressure cuff attached to the right arm. Both feet were strapped into the stepper with a support placed under the left leg to ensure complete relaxation of the limb. After 20 min rest and a stable baseline (identified by HR, VO_2_, blood pressure), blood velocity and diameter of the left common femoral artery were obtained to assess leg blood flow. As our aim was to isolate skeletal muscle blood flow, in some cases local leg cooling via wet towels was applied due to contamination of high skin blood flows (Limberg et al., 2020). Following acquisition of baseline parameters, participants commenced stepping exercise at the predefined workloads. After a steady state of exercise was reached (2 mins) during each workload, a 2‐min continuous measurement of blood velocity was taken (measurement 1) using vascular ultrasound (described below). The ultrasound probe was subsequently lifted from the limb for 5 s while the participants continued to exercise and then placed back onto the exercising limb for a repeat 2‐min measurement (measurement 2). Immediately following cession of exercise, diameter of the common femoral artery was obtained in B‐mode. Diameter measurements were not taken in the short interval when the probe was lifted away from the limb but were taken at a single time point at the end of each workload. The protocol was replicated for each workload with a minimum of 5 min rest interspersed between exercise bouts to allow cardiovascular variables to return to baseline (Measurement 1). Participants repeated the protocol on a separate day for assessment of between‐day reliability (Measurement 2, Figure 1).

### 2.4 Measurements

#### 2.4.1 Cardiovascular

Participants were instrumented with a three‐lead electrocardiogram (Tram‐rac, Solar 8000 M, GE‐ Marquette, USA) for continuous monitoring of heart rate (HR). At rest and during each workload, blood pressure was measured in duplicate, via electro‐sphygmomanometry (Tango, SunTechMedical Instruments Inc., USA) from a cuff placed on the right arm with a microphone placed over the brachial artery to detect Korotkoff sounds.

#### 2.4.2 Oxygen consumption

VO_2_ was measured continuously from expired gas collected through a mixing chamber which was connected to an independent gas analyzer (Moxus metabolic system, AEI Technologies Inc., USA), and ventilation from a pneumotachometer (Hans Roudolph Shawnee, USA).

#### 2.4.3 Femoral artery blood flow

Time‐average mean blood velocity (TAMV) and arterial diameter of the left common femoral artery were imaged using a 9‐MHz linear‐array Doppler probe (iE33, Philips, Netherlands). Blood velocity values from the iE33 probe were converted from an analogue to digital signal (Doppler audio converter, Penn State, Hershey, Pennsylvania, USA) (Herr et al., 2010) for accurate determination of blood flow. Measurements were taken below the inguinal ligament and approximately 2 cm above the bifurcation into the superficial femoral artery. Prior to all testing sessions, time was taken to identify the most appropriate area to obtain reliable Doppler signals during stepping exercise. Once marked, using an inedible ink pen, participants were asked to avoid washing the area until the repeat test had been performed to aid consistency between measurements days. Furthermore, basic ultrasound settings including depth, gain, power, and sample volume were pre‐set for the common femoral artery and after minor changes specific to each participant, were kept consistent across all measurement time points. The TAMV was obtained in pulse wave mode at an insonation angle of 60° and arterial diameter in B‐mode for 30 s. Common femoral blood flow was calculated from the product of arterial diameter and timed average mean blood velocity ([mean blood flow velocity × (π × (arterial diameter / 2)^2^)] ×60). Diameter of the femoral artery was analyzed offline using a custom designed wall tracking software (Coolbaugh et al., 2016).

### 2.5 Data analyses

All variables (except vascular diameter and blood pressure) were sampled at 0.4 kHz using the PowerLab data acquisition system (ADInstruments, UK) and analyzed with LabChart (Version 8, ADInstruments, UK). *Cardiovascular*. Mean arterial pressure was calculated by the sum of one third systolic and two thirds diastolic blood pressure and used to calculate femoral vascular conductance. *Oxygen consumption*. An estimation of oxygen consumption (STPD) was obtained according to the Haldane equation ([VE × FiO_2_ × ((1 – FeO_2_ + FeCO_2_) / FiN_2_)] – [(VE ×FeO_2_)]), where Fi and Fe are inspired and expired fractions of oxygen, carbon dioxide, and nitrogen, respectively, and VE is the volume corrected minute ventilation.

### 2.6 Statistical analyses

Validity of the workloads was assessed from plotting blood flow against oxygen uptake to deduce metabolic demand at each workload (i.e., metabolic flow coupling). Data from each of the individual measurements from (days 1 and 2) measurement 1 and 2 were all normally distributed. However, when the data were combined for correlation analysis it became non‐normally distributed. Therefore, measurements from all testing sessions were compared by repeated measures ANOVA with Sidak's multiple comparisons test. Moreover, a Spearman's correlation analysis was performed between muscle blood flow and systemic oxygen uptake.

### 2.7 Reliability

#### 2.7.1 Within session

For the within‐day assessment of technical reliability, blood flow of the two repeat measurements (1 vs. 2) were compared at each workload on both testing days (probe on and off) for assessment of intra‐rater reliability.

#### 2.7.2 Between‐session

For the between‐day comparison of technical and biological reliability, blood flow values for each workload were compared between days 1 and 2. The mean blood flow value obtained from measurement 1 and 2 from both trials were averaged for between day comparison. Reliability metrics such as the standard error of the estimate, coefficient of variation, Bland–Altman plot with limits of agreements, and linear regression analyses were performed (Bland & Altman, 1986). Moreover, cardiorespiratory variables within and between days were compared by repeated measures ANOVA with Sidak's multiple comparisons test. All data are expressed as mean ±standard deviation with statistical significance set at *p *≤ 0.05.

## 3 RESULTS

### 3.1 Workload intensity

**FIGURE 2:**
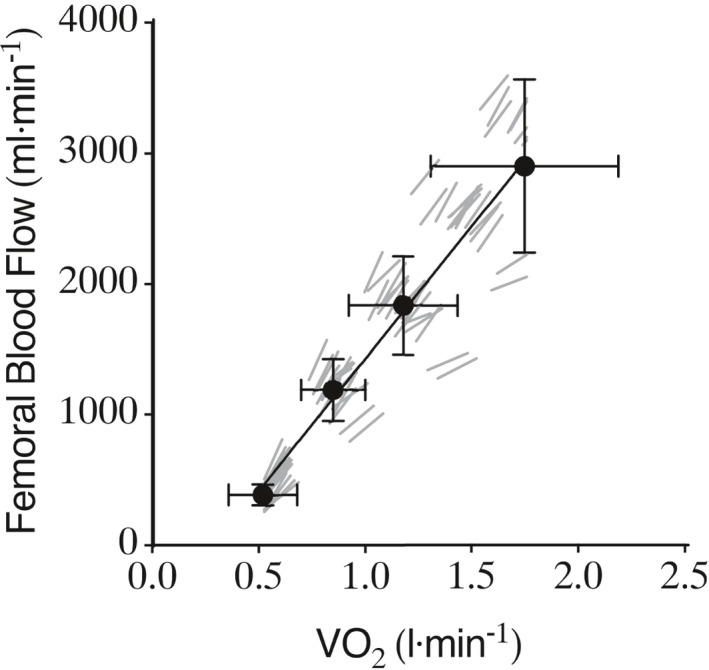
Femoral blood flow plotted against VO_2_ at rest and during different exercise intensities. Values represent means with standard deviation (n = 17). Abbreviations: WL, workload. P‐value represents results from one‐way ANOVA

The workloads performed during stepping exercise equated to an oxygen uptake of 0.85 l·min^−1^ (WL1), 1.18 l·min^−1^ (WL2), and 1.75 l·min^−1^ (WL3) (Figure 1) and the oxygen uptake was significantly different between the three workloads when compared within day (*p* = 0.0176; Table 1) and between days (*p* = <0.0001; Table 2) and produced a linear increase in femoral blood flow (Figure 2).

**TABLE 1:** Within‐session femoral blood flow reliability data for semi recumbent stepping exercise performed at three independent workloads (n = 17)

	Workload one (VO_2_, 0.85 l∙min^−1^)		Workload two (VO_2_, 1.18 l∙min^−1^)		Workload three (VO_2_, 1.75 l∙min^−1^)	
	Measurement 1	Measurement 2	Measurement 1	Measurement 2	Measurement 1	Measurement 2
FBF (ml∙min^−1^)	1238 ± 280	1228 ± 302	1898 ± 398	1876 ± 500	2902 ± 612	2908 ± 738
TEE (ml∙min^−1^)	45 (0.16)		146 (0.34)		148 (0.22)	
Lower CI (ml∙min^−1^)	35 (0.12)		114 (0.27)		115 (0.17)	
Upper CI (ml∙min^−1^)	64 (0.22)		207 (0.48)		209 (0.32)	
Inter‐class correlation	0.98		0.91		0.96	
CV (%)	4.1		8.2		5.3	

Abbreviations: VO_2_, oxygen consumption; FBF, femoral blood flow; TEE, typical error of the estimate; CI, confidence interval; CV, coefficient of variation. Trial 1 refers to the first measurement and trial 2 refers to the repeat measurement performed after the probe had been lifted away from limb (n = 17).

**TABLE 2:** Between day comparison of stepping exercise performed at three independent exercise workloads

	Workload one (VO_2_, 0.85 l∙min^−1^)		Workload two (VO_2_, 1.18 l∙min^−1^)		Workload three (VO_2_, 1.75 l∙min^−1^)	
	Day 1	Day 2	Day 1	Day 2	Day 1	Day 2
FBF (ml∙min^−1^)	1229 ± 299	1162 ± 290	1862 ± 442	1820 ± 435	2865 ± 671	2957 ± 803
TEE (ml∙min^−1^)	182 (0.78)		204 (0.52)		264 (0.38)	
Lower CI	141 (0.61)		158 (0.41)		205 (0.30)	
Upper CI	261 (1.13)		292 (0.75)		380 (0.55)	
Inter‐class correlation	0.65		0.81		0.89	
CV (%)	15.6		12.5		10.4	

Abbreviations: VO_2_, oxygen consumption; FBF, femoral blood flow; TEE, typical error of the estimate; CI, confidence interval; CV, coefficient of variation (n = 16).

### 3.2 Within‐session cardiorespiratory responses

Furthermore, HR, VO_2_, systolic, and mean arterial pressure progressively increased with exercise intensity, whereas, as expected, diastolic blood pressure did not change with the increase in exercise workloads (*p* = 0.5487) (Table 3). Importantly, there was no significant difference in any of the aforementioned variables between measurements one and two within day at all workloads (Table 3).

**TABLE 3:** Within‐session comparison of cardio‐respiratory variables

	Baseline	Workload one		Workload two		Workload three			
		Measurement 1	Measurement 2	Measurement 1	Measurement 2	Measurement 1	Measurement 2	Within‐session effect	Workload main effect
Heart Rate (beats·min ^−1^)	59 ± 8	74 ± 8	73 ± 8	83 ± 6	84 ± 6**	96 ± 9	99 ± 11**	0.0784	<0.0001
Systolic Blood pressure (mmHg)	119 ± 8	124 ± 10	125 ± 9	131 ± 9	132 ± 13**	148 ± 20	149 ± 22**	0.1933	<0.0001
Diastolic Blood pressure (mmHg)	63 ± 6	66 ±7	64 ± 8	66 ± 9	67 ± 9	66 ± 7	65 ± 8	0.2338	0.5487
Mean arterial pressure (mmHg)	81 ± 4	85 ± 6	86 ± 6	87 ± 8	88 ± 8**	93 ± 7	93 ± 9**	0.8989	<0.0001
VO_2_ (l·min^−1^)	0.51 ± 0.19	0.84 ± 0.16	0.86 ± 0.15	1.16 ± 0.23	1.21 ± 0.28*	1.76 ± 0.44	1.75 ± 0.46*	0.3097	0.0176

Within day comparison of measurements one (trial 1) versus measurement two (trial 2) (n = 17) for all variables except VO_2_ which was (n = 14). Baseline values were not included in statistical analysis. Within‐session main effect represents the comparison between test 1 and test 2 for each independent workload. The workload main effect represents a comparison between the mean of test one and two for each workload (*p ≤ 0.05, **p ≤ 0.01). Values are means with SD.

### 3.3 Between‐session cardiorespiratory responses

Similar to the within‐session data, all variables except diastolic blood pressure (*p* = 0.5729) demonstrated a significant increase with the progressive rise in exercise intensity (*p* = <0.0001). In addition, HR, VO_2_, diastolic, and mean arterial pressure were not different between days one and two for all workloads (Table 3). However, systolic blood pressure was significantly different between days for workload one (*p* = 0.0272) yet remained similar between days for workload two (*p* = 0.2821) and three (*p* = 0.4400).

### 3.4 Within‐session reliability

**FIGURE 3:**
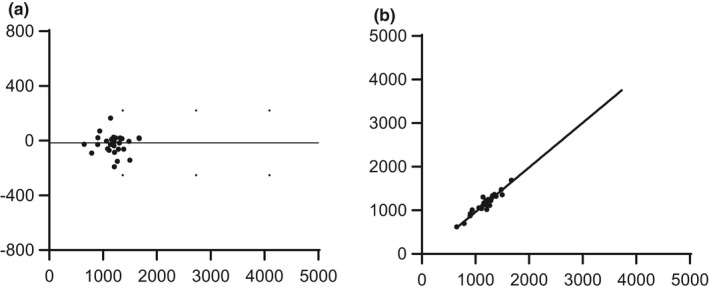
Within session reliability of femoral blood flow (FBF) during semi‐recumbent stepping exercise (n = 17). The agreement between trial one and trial two is shown in the Bland Altman plot (a), which contains the mean values of all within session data points from both days and the strength of the relationship in the scatter plot (b). Abbreviations: TE, typical error of estimate; CI, confidence interval; CV, coefficient of variation

The within‐session reliability for femoral blood flow was lowest at the lightest exercise workload, producing a typical error of 45 ml·min^−1^ with mean blood flow equaling 1233 ± 291 ml·min^−1^. As exercise intensity increased the typical error also increased and was 146 ml·min^−1^ for workload two, with mean blood flow equaling 1887 ± 449 ml·min^−1^ and 148 ml·min^−1^ for workload three, with blood flow equal to 2905 ± 675 ml·min^−1^ (Table 1). The coefficient of variation was also lowest for WL1 (4.1%) and increased to 8.2% for WL2 but was reduced to 5.3% for WL3 (Table 1). The within‐session measurement bias calculated from the mean of the difference between repeat measurements was −16 ± 121 ml·min^−1^ and the upper and lower limits of agreement were 221 ml·min^−1^ and −253 ml·min^−1^ (Figure 3a).

### 3.5 Between‐session reliability

**FIGURE 4:**
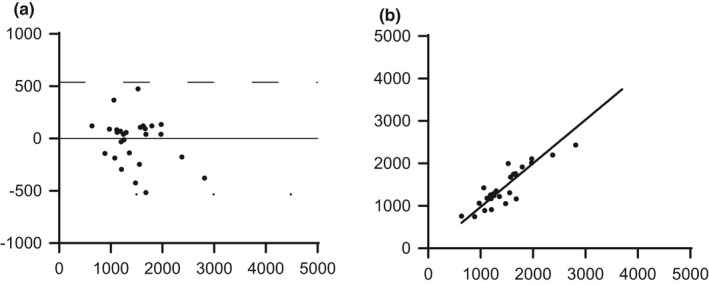
Between session reliability of femoral blood flow (FBF) during semi‐recumbent stepping exercise (n = 16). The agreement between mean values on day one is plotted against mean values from day two and shown in the Bland Altman plot (a) and the strength of the relationship in the scatter plot (b). Abbreviations: TE, typical error of estimate; CI, confidence interval; CV, coefficient of variation

The between‐session reliability for femoral blood flow produced a higher typical error and coefficient of variation, as expected compared to the within day measurements. The typical error increased with the rise in exercise intensity and like the within‐session data was lowest for WL1 (182 ml·min^−1^), increasing to 204 ml·min^−1^ for WL2 and 264 ml·min^−1^ for WL3 (Table 2). In contrast, the coefficient of variation decreased with increasing workload and was lowest for WL3 (10.4%), increasing to 12.5% for WL2 and 15.6% for WL1 (Table 2). The between‐session measurement bias calculated from the mean of the difference between measurements across both days were 29 ± 344 ml·min^−1^ and the upper and lower limits of agreement were 702 ml·min^−1^ and −645 ml·min^−1^ (Figure 4a).

### 3.6 Within‐session diameter and time average mean blood velocity

Both common femoral artery diameter (*p* = 0.0137) and TAMV (*p* = <0.0001) increased significantly with the rise in exercise intensity. However, TAMV was not significantly different between measurements at each independent workload (Table 4).

**TABLE 4:** Within‐Session changes in femoral artery diameter and blood velocity

	Baseline	Workload one	Workload two	Workload three	ANOVA P‐value
Within session					
Arterial diameter (mm)	0.961 ± 0.111	0.988 ± 0.116*	0.995 ± 0.108*	1.001 ± 0.097*	0.0137

		Measurement 1	Measurement 2	Measurement 1	Measurement 2	Measurement 1	Measurement 2	Within‐ session main effect	Workload main effect	Interaction
TAMV (cm·s^−1^)	8.88 ± 2.89	27.4 ± 6.98	27.0 ± 7.14	40.9 ± 6.93	39.8 ± 6.32**	62.2 ± 9.55	61.0 ± 10.44**	0.1115	<0.0001	0.6192

Note: Baseline values for velocity were not included in the repeated measures ANOVA (n = 17). Within‐session main effect represents the comparison between trial one and two for each independent workload. Diameter values represent measurements taken after the second recordings of blood velocity had been undertaken. For diameter, stars signify the difference between workloads (workload main effect) and for TAMV, stars represent a comparison between the mean of trial one and two for each workload (*p = <0.05, **p = ≤0.01). Values are means with SD.

### 3.7 Between‐session diameter and time average mean blood velocity

Arterial diameter was not significantly different between measurements (day 1 vs. day 2) for each independent workload (*p* = 0.6690), however diameter significantly increased with the rise in exercise intensity (*p* = <0.0006). TAMV was also not significantly different between measurements (day 1 vs. day 2) for each independent workload (*p* = 0.3282), however, did significantly increase with the rise in exercise intensity (*p* = <0.0001).

## 4 DISCUSSION

The primary aim of this study was to validate a model of dynamic lower limb exercise that resembled traditional forms of exercise whereby skeletal muscle blood flow could be reliably quantified noninvasively via ultrasonography. During graded stepping exercise, common femoral artery blood flow was linearly related to oxygen uptake with a within‐session technical error of 5.8% over a range of mild to moderate exercise intensities and a between‐session reliability of 12.7%.

The linear increase in skeletal muscle blood flow with whole‐body oxygen uptake during graded sub maximal stepping exercise matched the invasive response of muscle blood flow during cycling (Proctor et al. 2003), therefore enabling assessment of reliability at three independent workloads. When considering individual workloads, we found the lowest within‐session coefficient of variation at the lowest exercise workload (WL1) (4.1%) and slightly higher values at WL2 (8.2%) and WL3 (5.3%). This was expected due to greater limb and extraneous bodily movement that occurs during higher intensity exercise, which increases the difficulty of obtaining stable recordings (Table 1). In contrast, the opposite trend was observed for the between day assessment of reliability with the highest coefficient of variation reported at the lowest workload (WL1) (15.6%), compared with WL2 (12.5%) and WL3 (10.4%). Furthermore, the coefficient of variation was lower for the within‐session measurements compared to the between‐session values for all workloads when combined and compared individually (Tables 2 and 3). This result was expected due to higher random error associated with greater between day biological variations that may influence muscle blood flow.

In the current study, the within‐session reliability reflects the measurement of technical error, as exercise was performed in a steady state with only a few seconds in between measurements. The measurement error depicted in the Bland–Altman plot was −16 ± 121 ml·min^−1^ for the within‐session data. The between‐session reliability was conducted over a period of at least 1 week and thus reflects not only technical error by the operator, but biological variability. The between‐session blood flow values demonstrated a slightly larger mean difference of 29 ± 344 ml·min^−1^. However, the greater SD (344 ml·min^−1^) meant a greater upper and lower confidence interval range (−645 to 702 ml·min^−1^), thereby accounting for the greater spread in the data.

Based on these data, the absolute typical error of the estimate was 45 ± 0.16; 146 ± 0.34 & 148 ± 0.22 ml·min^−1^ across the three workloads within‐session and 182 ± 0.78; 204 ± 0.52 & 264 ± 0.38 ml·min^−1^ between sessions. When these values are placed in the context of ultrasound blood flow measurement during exercise (Walther et al., 2006), (MacDonald et al., 1998), which range from 1165 to 2908 ml·min^−1^, the size of the typical error is relatively small even for the between‐ session comparison, despite a modest coefficient of variation (~12.7%). This is especially true considering blood flow values can reach upward of 7 l·min^−1^ as measured by ultrasound during knee extensor exercise (Rådegran, 1997).

### 4.1 Comparison with existing data

Rådegran, (1997) was the first to report within day coefficient of variation of ~6% using Doppler ultrasound during knee extensor exercise. Walther et al. (2006) succeeded this, reporting between day values of ~11.1%, ~7.1%, and ~12.1% using the same exercise model performed at three increasing workloads. Our within day (~4.1%, ~8.2%, and ~5.3%) and our between day (~15.6%, ~12.5%, & ~10.4%) values align with previous data despite the use of a different exercise modality. However, Choo et al. (2017) performed a highly comparable study which measured common femoral blood flow pre‐ and immediately post (3 min) repeated high intensity cycling bouts. They reported a within day coefficient of variation of ~10.5%, which like our data is slightly higher than those reported during knee extensor exercise and thus appears to suggest that isolated single knee extensor exercise provides an easier methodological approach for capturing blood flow due to the fixed range of motion and rhythmic nature of contraction.

### 4.2 Influence of diameter and velocity

It is important to acknowledge that blood flow reliability scores are based on changes in velocity and arterial lumen diameter. Typically, it is common for most studies using ultrasound to perform measurements of diameter during end diastole (Shoemaker et al., 1997), (Hansen et al., 2020; Van Beekvelt et al., 2001), with some studies reporting changes with exercise (Shoemaker et al., 1997), (Amundsen et al., 2002), (Christiansen et al., 2020) and others not (Walløe & Wesche, 1988), (Eriksen et al., 1990), (Osada et al., 1999). Measurement error could certainly account for such differences, especially given that changes in diameter are so small (0.03–0.04 mm); however the development of wall tracking software removes an element of human measurement error and appears to demonstrate that changes in diameter typically are present during systole (Woodman et al., 2001). Indeed, wall tracking software has been reported to produce greater reproducibility compared to classical manual measurements (Woodman et al., 2001) (Green et al., 2002), (Beux et al., 2001). Our measurements were obtained with custom designed wall tracking software and demonstrated that the common femoral diameter did increase with the rise in exercise intensity (Tables 4 and 5). Moreover, we demonstrated that the common femoral diameter was similar between days at each workload which provides support for the reliability of ultrasound‐based measurements of femoral diameter with exercise and demonstrates good between day confirmation of dilation during lower body exercise. After exercise cessation, common femoral velocity decreases rapidly and therefore to accurately obtain absolute measurements of blood flow relative to oxygen uptake, measurements should be made during exercise. Our data demonstrates that during exercise there is no difference in velocity within (*p* = 0.1115) and between (*p* = 0.3283) session for all workloads, thus supporting the accuracy of our reliability scores.

**TABLE 5:** Between‐Session changes in femoral artery diameter and blood velocity

	Baseline		Workload one		Workload two		Workload three				
Between session	Day 1	Day 2	Day 1	Day 2	Day 1	Day 2	Day 1	Day 2	Between session main effect	Workload main effect	Interaction
Arterial diameter (mm)	0.971 ± 0.110	0.981 ± 0.129	0.988 ± 0.116	0.989 ± 0.138**	0.995 ± 0.108	0.990 ± 0.127**	1.001 ± 0.097	1.010 ± 0.115**	0.6715	0.0006	0.3228
	Day 1	Day 2	Day 1	Day 2	Day 1	Day 2	Day 1	Day 2	Between Session Main effect	Workload Main effect	Interaction
TAMV (cm·s^−1^)	8.88 ± 2.89	9.13 ± 2.55	27.2 ± 7.04	26.1 ± 5.54**	40.4 ± 6.44	39.5 ± 7.42**	61.1 ± 9.91	60.8 ± 11.57**	0.3282	<0.0001	0.9057

Note: Baseline values were included in analysis for both parameters (n = 16). Between‐session main effect represents the comparison between day one and two for each independent workload. The workload main effect represents a comparison between the mean of day one and two for each workload (*p = <0.05, **p = ≤0.01). Values are means with SD.

### 4.3 Applicability

The premise of the study was to validate a model of dynamic lower limb exercise that resembled physiological changes associated with traditional forms of exercise, with the intension of applying the model as an integrative assessment of multiple physiological parameters that coordinate the response to exercise in elderly and/or clinical populations. With regards to the oxygen cascade, several acceptable approaches exist to measure respiratory and central cardiac limitations, such as cardiac output via inert gas rebreathing, during dynamic exercise. Yet, the assessment of muscle blood flow has been technically challenging during such exercise. To date, most previous studies have used isolated knee, (Walther et al., 2006) or elbow extensor (Green et al., 2005) exercise to quantify blood flow patterns during movement. The modality has been successfully adopted as a safe mode of exercise in patients with chronic heart failure by limiting excessive strain on central cardiopulmonary regions (Esposito et al., 2018), while facilitating peripheral adaptions (Richardson, 2004). However, the modality of exercise is unable to elicit the same global physiological response of continuous dynamic exercise which includes, an increase in heart rate and stroke volume, coupled with changes in muscle sympathetic nerve activity and vasoconstriction in non‐active skeletal muscle. Furthermore, the model can also integrate the use of environmental stressors such as hypoxia, heat and cold to potentially mimic exercise performed in more real‐life settings. In the current model, light workloads produced substantial skeletal muscle vasodilation, which was largely independent of central limitations as measured by changes in heart rate and stroke volume at similar exercise intensities (Amin et al., 2021). Thereafter, active skeletal muscle vasodilation continued to increase alongside VO_2_, whereby stroke volume also increased as did resistance in the non‐active forearm (Amin et al., 2021), suggestive of sympathetically mediated vasoconstriction (Hansen et al., 2020). Together, these data paint a picture of the classical physiological responses observed during large dynamic lower limb exercise (Laughlin, 1999) and highlight that this model can be used to noninvasively assess several components of the oxygen cascade, including muscle blood flow and oxygen delivery using an experimental approach.

### 4.4 Limitations

The main limitation of the current study was that the workloads were based on absolute VO_2_ and percentage of maximal heart rate rather than watts, which at that time, was not possible due to the original Ergopect steppers air pressure operating principle. Over the three selected workloads, the mean physiological responses (heart rate, blood pressure, and VO_2_ data (Tables 3 and 6) were very well controlled, with no differences within‐ or between‐session. Yet, the higher coefficient of variation in skeletal muscle blood flow between days, may be partly due to slightly greater variability in VO_2_ (CV: WL1, 22.5%, WL2, 16.7%, and WL3, 11.7%) caused by differences in external workload (watts). Nonetheless, despite this limitation, between‐session variability was still acceptable (WL1, 15.6%, 182, ml∙min^−1^; WL2, 12.5%, 204, ml∙min^−1^; and WL3, 10.4%, 264, ml∙min^−1^) and it can only be imagined that these reliability statistics will improve with the new model that precisely control watts (http://www.ergospect.com/). We should also draw the reader's attention to the fact that femoral diameters were obtained immediately after each exercise bout, therefore, our values for blood flow may slightly underestimate the true response observed during exercise (Walther et al., 2006). However, in opposition to changes in velocity (seconds), changes in conduit arterial diameter are typically protracted, even with very rapid changes in shear stress (Pyke & Tschakovsky, 2005), thus the aforementioned observation (Walther et al., 2006) maybe a technical rather than physiological difference. Thus, in our opinion, it is important to focus on high‐resolution velocity recordings during exercise and the diameter measured in the second preceding exercise are reflective of the steady‐state condition at the end of each workload. We also acknowledge that our reliability scores are highly specific to the three distinct workloads which were all submaximal in our young healthy participants; therefore, we must be cautious in applying our results to other forms of exercise and extrapolating the reliability scores to higher exercise intensities. While the chosen workloads reflect activities of daily living and changing physiological states (local skeletal muscle hyperemia with and without large changes in systemic hemodynamics), higher workload up to aerobic capacity would have been interesting. Unfortunately, at the time, stability and smoothness of the stepping stroke was not optimal at very high watts in the prototype device, but now is markedly improved and accurate measurements of blood flow at very high watts maybe possible with the newer model. While we did not include a comparison of sex, it is highly unlikely that sex‐based differences in vessel geometry / morphology etc. would have impaired the reliability of this technique. In contrast, these data cannot be applied to clinical populations, with limb abnormalities or obese individuals where fat mass may make ultrasonography more challenging. Finally, while our model replicates traditional aerobic exercise and supports our objective of identifying skeletal muscle blood flow limitations within the oxygen cascade, we acknowledge that it may not provide the optimal form of exercise for vascular adaptation. Generating high blood flow and favorable shear stress profiles can be achieved with isolated limb exercise and in clinical populations this model may serve as a more appropriate method for the early stages of rehabilitation and vascular adaptation (Tinken et al. 2010).

**TABLE 6:** Between‐session comparison of cardio‐respiratory variables

	Baseline	Workload one		Workload two			Workload three			
		Day 1	Day 2	Day 1	Day 2		Day 1	Day 2	Between‐ session main effect	Workload main effect
Heart Rate (beats·min ^−1^)	59 ± 8	74 ± 10	73 ± 8	84 ± 9	84 ± 9**		100 ± 11	95 ± 10**	0.1889	<0.0001
Systolic Blood pressure (mmHg)	119 ± 8	127 ± 10	122 ± 10†	134 ± 18		129 ± 11**	146 ± 17	151 ± 25**	0.0346	<0.0001
Diastolic Blood pressure (mmHg)	63 ± 6	66 ± 6	63 ±9	67 ± 8	66 ± 11		66 ± 6	65 ± 10	0.2927	0.5729
Mean arterial pressure (mmHg)	81 ± 4	86 ± 5	82 ± 7	89 ± 9	86 ± 9**		94 ± 9	92 ± 8**	0.0940	<0.0001
VO_2_ (l·min^−1^)	0.51 ± 0.19	0.85 ± 0.19	0.86 ± 0.18	1.25 ± 0.31	1.12 ± 0.24**		1.82 ± 0.49	1.69 ± 0.43**	0.1313	<0.0001

Note: Between day comparison of cardio‐respiratory parameters (day 1 vs. day 2), n = 16 for all variables except VO_2_ which was n=14. Baseline values were not included in statistical analysis. Between‐session main effect represents the comparison between‐day one and two for each independent workload (^†^ *p* ≤ 0.05). The workload main effect represents a comparison between the mean of day one and two for each workload (*p ≤ 0.05, **p ≤ 0.01). Values are means with SD.

## 5 CONCLUSION

Our measurement of skeletal muscle blood flow using Doppler ultrasound during dynamic continuous semi‐recumbent stepping exercise produced a within‐session coefficient of variation of 5.8% and a between‐session coefficient of variation of 12.7% during exercise performed at three distinct workloads. Our within‐session values provide confidence in our ability to accurately measure flow up to an intensity of approximately 50% of maximal heart rate in young healthy individuals. These data support our translational aim of utilizing the model as an integrative assessment of exercise intolerance in multiple populations.

## DISCLOSURES

No conflict of interests, financial, or otherwise are declared by the authors.

## AUTHOR CONTRIBUTIONS

J.S.L, F.E.D, and S.B.A conceived and designed the study. F.E.D, S.B.A, H.M, and K.M performed experiments and recruited the participants. S.B.A and F.E.D analyzed the data. J.S.L, J.P.M, and S.B.A prepared the manuscript. All authors assisted with editing and approved the final manuscript.
